# Nutrition and Physical Activity in Nonalcoholic Fatty Liver Disease

**DOI:** 10.1155/2016/4597246

**Published:** 2015-12-07

**Authors:** Claudia P. Oliveira, Priscila de Lima Sanches, Erlon Oliveira de Abreu-Silva, Aline Marcadenti

**Affiliations:** ^1^Department of Gastroenterology, School of Medicine, University of Sao Paulo (USP), 255 Dr. Enéas de Carvalho Aguiar Avenue, Cerqueira César, 05403-900 Sao Paulo, SP, Brazil; ^2^Postgraduate Program in Nutrition, Federal University of Sao Paulo (UNIFESP), 630 Marselhesa Street, Vila Clementino, 04020-060 Sao Paulo, SP, Brazil; ^3^Division of Interventional Cardiology and Postgraduate Program in Cardiology, Federal University of Sao Paulo (UNIFESP), 715 Napoleao de Barros Street, Vila Clementino, 04024-002 Sao Paulo, SP, Brazil; ^4^Department of Nutrition, Federal University of Health Sciences of Porto Alegre (UFCSPA), 245 Sarmento Leite Street, Centro Histórico, 90050-170 Porto Alegre, RS, Brazil; ^5^Postgraduate Program in Health Sciences: Cardiology, Institute of Cardiology of Rio Grande do Sul (IC/FUC), 395 Princesa Isabel Avenue, Santana, 90040-371 Porto Alegre, RS, Brazil

## Abstract

Nonalcoholic fatty liver disease (NAFLD) is the most common liver disease worldwide and it is associated with other medical conditions such as diabetes mellitus, metabolic syndrome, and obesity. The mechanisms of the underlying disease development and progression are not completely established and there is no consensus concerning the pharmacological treatment. In the gold standard treatment for NAFLD weight loss, dietary therapy, and physical activity are included. However, little scientific evidence is available on diet and/or physical activity and NAFLD specifically. Many dietary approaches such as Mediterranean and DASH diet are used for treatment of other cardiometabolic risk factors such as insulin resistance and type-2 diabetes mellitus (T2DM), but on the basis of its components their role in NAFLD has been discussed. In this review, the implications of current dietary and exercise approaches, including Brazilian and other guidelines, are discussed, with a focus on determining the optimal nonpharmacological treatment to prescribe for NAFLD.

## 1. Introduction

**Figure 1:**
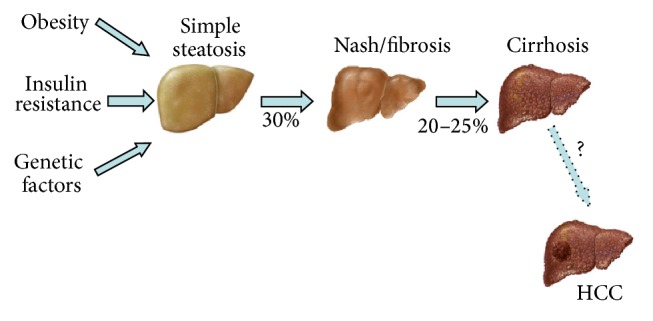
Spectrum of NAFLD. Obesity, genetic factors, and insulin resistance (IR) contribute to fat deposition in the liver. Approximately 30% of patients with NAFLD have NASH, which involves the presence of various degrees of inflammation and fibrosis, and can progress to cirrhosis in 20–25% of cases within 20 years. An unknown percentage of cases develop HCC. Adapted from Trauner et al., 2010 [9].

The term nonalcoholic fatty liver disease (NAFLD) is used to define a large spectrum of diseases related to hepatic fat deposition: hepatic steatosis (HS) is diagnosed when there is only fat accumulation; nonalcoholic steatohepatitis (NASH) is characterized when besides steatosis, there is inflammation, ballooning and moderate fibrosis; and the evolution from NASH to cirrhosis and hepatocellular carcinoma (HCC) [1, 2] (Figure 1). Even though it presents similar morphological findings when compared to the alcohol-induced lesion, NAFLD occurs in subjects with no significant alcohol consumption (<20–40 g/day) [3].

Currently, NAFLD is one of the more frequent causes of liver disease with an estimated prevalence of 20 to 30% in developed countries. Its incidence has significantly increased during the past years as a consequence of the raise of obesity in the Western world, due, mainly, to a sedentary lifestyle and inappropriate eating habits [2]. It is estimated that one-quarter of the American population is overweight, and 80% of these have NAFLD [4]. Among subjects with class III obesity [body mass index (BMI) BMI > 40 kg/m^2^] its incidence is 90%. When it comes to NASH, the prevalence is around 2-3% [4].

NAFLD is highly prevalent in patients with diabetes mellitus and increasing evidence suggests that patients with type-2 diabetes (T2DM) are at a particularly high risk for developing the progressive forms of NAFLD, NASH, advanced liver fibrosis, hepatocellular carcinoma development, and liver-related mortality [5]. In these individuals, prevalence of NAFLD ranges from 42.6% [6] to 79% [7]. It is noteworthy that these numbers vary from country to country and that nearly 13% of cases have already presented signs of cirrhosis [7]. In a sample of American persons with T2DM and normal serum levels of aminotransferases, prevalence of NAFLD and NASH was 76% and 56%, respectively [8].

Since there is no proven pharmacologic treatment for NAFLD, the aim of this review is to discuss the role of the diet and physical exercise as a nonpharmacologic treatment for NAFLD related to T2DM, metabolic syndrome, and other cardiometabolic risk factors, based on current evidence and guidelines.

## 2. Risk Factors and Pathophysiology

NAFLD is associated with the components of metabolic syndrome (MS): T2DM, insulin resistance (IR), hypertension (HTN), and, mainly, abdominal (visceral) obesity and dyslipidemia (hypertriglyceridemia, low levels of high density lipoprotein (HDL), and/or high levels of low density lipoprotein (LDL)). Besides, it also can be associated with surgical procedures (i.e., jejune-ileum bypass), protein-caloric malnutrition, prolonged parenteral nutrition, drug addiction, medications, toxins, and endocrine pathologies [10, 11].

Presently, most authors believe in the “multiple hits” theory, in which IR plays a central role as the inciting event for fatty acid accumulation in the hepatocyte: “the first hit.” The rationale is that IR favors lipogenesis and inhibits lipolysis, which drastically increases the influx of fatty acids in the liver. This, in turn, is followed by an increase in oxidative stress, endoplasmic reticulum and mitochondrial dysfunctions, and chronic endotoxemia: “multiple hits.” Once steatosis takes place, the liver becomes vulnerable to these “hits,” which leads to hepatocellular damage, inflammation, and fibrosis [12–14].

A more detailed look shows that IR is a key factor for stimulating the movement of free fatty acids (FFA) to *β*-oxidation, which depletes the mechanism of oxidative phosphorylation and the production of adenosine triphosphate (ATP) and incites the release of reactive oxygen species (ROS); this, in turn, leads to cell injury and activates the cascade to fibrosis. Similarly, IR also blocks the exportation of triglycerides from the hepatocyte by degrading and preventing the production of very low density lipoproteins (VLDL) [15, 16].

In the context of NAFLD, the excessive influx of FFA is directed towards the hepatocytes' mitochondria in order to be oxidized, which promotes mitochondrial dysfunction by depletion of *β*-oxidation and leads to overproduction of ROS and increase of hepatic oxidative stress. In this scenario, of mitochondrial oxidation collapse, there is activation of alternative pathways (i.e., microsomes, peroxisomes) resulting in additional formation of ROS; this is possible because the balance between formation of ROS and antioxidant intracellular defenses is broken [17].

The byproducts of lipid peroxidation and cyclic formation of ROS are important proinflammatory agents that seem to activate hepatic stellate cells (HSC), thus leading to hepatic fibrosis [18, 19]. In this manner, progression from steatosis to NASH and fibrosis may occur by three different mechanisms: lipid peroxidation, induction of proinflammatory cytokines, and induction of Fas ligands [20].

Diabetes and NAFLD have mutual pathogenetic mechanisms and it is possible that genetic and environmental factors interact with metabolic derangements to accelerate NAFLD progression in diabetic patients [5].

Intestinal microbiome has an effect on obesity and RI, as well as hepatic fat content [21]. Patients with NAFLD and T2DM have significant changes in their microbiota composition and consequently higher prevalence of decompensated dysbiosis (about 72%) [22]. Individuals with obesity or NAFLD have more small intestinal bacterial overgrowth, which have been related (together with the intestinal permeability) to the degree of hepatic steatosis. Possible mediators of the link between the gut microbiome and the host include choline and endotoxins [21]. Choline deficiency, which can exist without a dietary deficiency, may be involved in the development of NAFLD and NASH [19]: high fat diets can induce an intestinal microbiota that converts dietary choline into methylamines, resulting in decreased levels of phosphatidylcholine. Phosphatidylcholine is important for the production of VLDL; thus, choline deficiency secondary to the intestinal microbiome may result in lower hepatic secretion of VLDL, with consequent triglyceride accumulation in hepatocytes [21, 23]. Intestinal permeability may lead to translocation of bacteria, increasing bacterial products such as lipopolysaccharide (LPS) and bacterial DNA. Subjects with NAFLD and increased intestinal permeability have increased hepatic exposure to endotoxins* via* the portal vein and it may lead to liver inflammation. Intestinal dysbiosis can also induce colonic inflammation, which accelerates the progression of simple steatosis to NASH. [21, 24].

Studies suggest that NAFLD can be associated with “idiopathic” cases of HCC. Most molecular events that lead to HCC need better clarification, but the main steps to cancer development (initiation, promotion, and progression) correlate with NAFLD physiopathology. Obesity is related to IR and augmentation of insulin-like growth factors, which act as a mitogen to stimulate cellular growth; in addition, obesity is associated with hyperestrogenemia, which is also implicated in the proliferation of hepatocytes. Increased production of ROS and DNA oxidative injury may also contribute to HCC development [25].

## 3. Diagnosis

NAFLD can occur at any age, although its prevalence is higher in the fourth and fifth decades. It is equally distributed between genders, but women have a tendency to develop advanced forms of the disease. Subjects with NAFLD are generally asymptomatic and find out about the disease because of elevated aminotransferases (ALT, AST) or during an abdomen ultrasonography (US) exam. Actually, fatty liver disease is an incidental finding during check-up exams or in the evaluation of persons with severe obesity, T2DM, and/or HTN (all these conditions are known risk factors for NAFLD). When present, symptoms are vague like right upper quadrant discomfort, fatigue, and dyspepsia; nausea, anorexia, and pruritus are rarely referred to by the patients.

On physical exam, subjects with NAFLD are frequently overweight (BMI > 25 kg/m^2^) and/or viscerally obese and have HTN. Hepatomegaly can be present at the time of diagnosis in 50% of patients with NAFLD (Table 1). Hepatic failure and portal hypertension stigma are less frequent than in other chronic liver diseases, although splenomegaly can be present in 25% of cases.* Acanthosis nigricans* (skin hyperpigmentation in dark plaques) is recognized as a clinical marker of IR and T2DM and can be identified in persons with NAFLD, mainly children.

Typically, patients with NAFLD have high levels of alanine aminotransferase (ALT), usually less than four times the upper normal limit. As a matter of fact, NAFLD is the most common cause of persistent ALT elevation. Nevertheless, in some cases, even in the presence of histological evidence of steatohepatitis, enzymatic levels can be normal. The other aminotransferase, aspartate aminotransferase (AST), has a milder elevation than ALT in NAFLD (different from what happens in alcoholic steatohepatitis) and can also be elevated in cirrhosis. It is noteworthy that half of the individuals with NAFLD will have concomitant high levels of gamma-glutamyl transferase (GGT).

Elevation of serum glucose, cholesterol (and its fractions), and triglycerides is frequently seen in cases of NAFLD. Routinely, hepatitis B and hepatitis C, autoimmune hepatitis, Wilson's disease, hemochromatosis, and alfa-1 antitrypsin deficiency must be excluded.

Although no noninvasive imaging method is able to distinguish simple steatosis to NASH or indicate the degree of liver fibrosis, US, computed tomography (CT), and magnetic resonance imaging (MRI) of the abdomen have been used as diagnostic tests for NAFLD. For its availability in most centers, low cost, and similar sensitivity to CT and MRI, US is the most used imaging method to detect NAFLD; however, it is a test that depends on the operator's experience. US sensitivity and specificity in detecting fatty infiltration decrease with increasing BMI, ranging from 49 to 100% and from 75 to 95%, respectively.

An innovative method that aims to quantify more precisely the liver fatty infiltration is the magnetic resonance spectroscopy [26]. Another interesting technique involves the use of a transducer placed between the intercostal spaces, which measures the speed at which a transient elastic shear wave travels through the liver [27, 28]. Fibrosis promotes liver solidification, increasing the travel speed of the shear waves; when compared to other noninvasive methods, FibroScan was at least as accurate as other methods and able to predict severe fibrosis. Although it is easy to apply, there are technical problems which can impair the screening of some individuals, such as morbid obesity and subjects who had small intercostal spaces; it is suggested that the best approach may be to use FibroScan together with other indexes or biomarkers [27, 28]. In fact, the use of liver elastography associated with the controlled attenuation parameter (CAP), which is a software that detects the amount of the fat in the liver, may help in the diagnosis of steatosis [29].

## 4. Nutritional and Dietetic Recommendations in NAFLD

The influence of diet composition in terms of macronutrients values on the development and progression of fatty liver diseases is not clear, and there seems to be some differences according to gender. Among Oriental men, a diet composed of a higher protein and cholesterol levels was associated with increased risk of alcoholic fatty liver disease (AFLD), while a diet rich in carbohydrates and sweets was protective for this condition. In women, a diet with high carbohydrate and sweets consumption was related to risk for NAFLD. In this survey, which used abdominal US for fatty liver disease diagnosis, there was no association between a dietary pattern and NAFLD in men [30].

In general, dietetic recommendations for NAFLD are the same as those for the different conditions known to be risk factors for it, that is, obesity, IR/T2DM, dyslipidemia, and HTN.

### 4.1. Weight Loss

Interventions promoting weight loss are frequently necessary in patients with NAFLD. Obese persons with NASH often consume higher amounts of calories, saturated fat, and cholesterol and lower amounts of polyunsaturated fatty acids, fibers, and vitamins C and E when compared to healthy individuals. Considering the metabolic changes induced by inadequate alimentary habits and weight gain, studies have shown that weight loss can not only reduce the volume of hepatic fat but also attenuate NASH-related inflammation and fibrosis [31].

A study evaluating dietetic modulation (with reduced caloric and saturated fat intake) as exclusive form of treatment for NAFLD in 31 subjects showed that, after 6 months, those who were considered adherent to the protocol (loss ≥ 5% of baseline weight) had lower BMI, waist circumference, ALT and GGT levels, visceral fat and hepatic density (analyzed by computed tomography), and HOMA-IR index (Homeostasis Model Assessment-Insulin Resistance). The diet protocol consisted of caloric restriction (500–1000 kcal/day) and prespecified amounts of nutrients (55% carbohydrates, 15% proteins, 30% lipids (8–10% saturated fatty acids, >15% monounsaturated fatty acids, >10% polyunsaturated fatty acids, and <300 mg/day cholesterol), and 20–30 g of fibers/day) [32].

A survey with 31 individuals with overweight/obesity (BMI 25–40 kg/m^2^) and biopsy-detected NASH evaluated the effect of dietetic intervention (1,000–1,500 kcal/day, 25% lipids; other orientations according to the American Heart Association, American Diabetic Association, and American College of Sports Medicine) for 48 weeks. Compared to control group (standard orientations), those on the intervention group lost more weight (9.3% versus 0.2%) and had a reduction on the* NAFLD Activity Score (NAS)*. It is noteworthy that those who lost ≥7% of baseline weight had a significative improvement on steatosis, lobular inflammation, ballooning, and NAS [33].

According to the American Gastroenterological Association (AGA), loss of 3–5% of baseline weight is necessary to improve steatosis, but a more substantial loss (>10%) may be necessary when necroinflammation is present [34].

### 4.2. Carbohydrates

Excessive ingestion of carbohydrates may be prejudicial to subjects with NAFLD, and this high intake seems to be related to the inflammatory state and disease progression. Diets with low levels of carbohydrates, low-carb (<45% of carbohydrates/day), show positive results regarding weight loss, reduction of intrahepatic triglyceride content, and improvement of metabolic parameters in obese subjects [35]. A meta-analysis evaluated solely randomized trials comparing low-carb and low-fat (≤25% lipids/day) diets in 2,800 persons; it demonstrated a significant reduction of triglycerides and increase of HDL with the low-carb diet [36].

However, in animal models, the maintenance of low-carb diets for long periods stimulates the development of NAFLD and glucose intolerance [35]; in humans, it is associated with discrete increments of total and LDL cholesterol [36]. Compared to diets with no carbohydrate restriction, low-carb diets seem to be more effective in terms of short-term weight loss (6 months), but this difference is not sustained after a year. Interestingly, a severe and quick weight loss may lead to disease progression in some patients with NAFLD [35].

In persons with this condition, modulation of the sort of carbohydrates seems to be more important than the amounts. Diets with high fructose content (added to food, mainly as corn syrup) are associated with development of MS and NAFLD; the ingestion of fructose stimulates* de novo* lipogenesis (resynthesis of fatty acids) and inhibits leptin secretion, which contributes to reducing satiety and increasing caloric intake [37]. However, a meta-analysis which evaluated both clinical trials and observational studies with adults and children showed that the apparent association between indexes of liver health (i.e., liver fat, hepatic* de novo* lipogenesis, ALT, AST, and GGT) and fructose or sucrose intake appear to be confounded by excessive energy intake. Besides, authors suggest that the available evidence is not sufficiently robust to draw conclusions regarding effects of fructose or sucrose consumption on NAFLD [38].

Recommendations of carbohydrate intake for persons with T2DM, dyslipidemia, and MS vary from 45 to 60% of daily total caloric ingestion (↓ 10% from caloric sweetener foods) mainly from fruits, vegetables, whole grains, legumes, and low-fat milk [39, 40].

### 4.3. Proteins

Little is known about the effect of quantity, quality, and composition of proteins in the development and treatment of NAFLD. In Brazilian subjects, a high protein-hypocaloric diet (1,200–1,400 kcal/day, 35% of mixed animal and vegetable protein) was associated with improvement of lipid profile, glucose homeostasis, and liver enzymes in NAFLD independently of BMI decrease or body fat mass reduction [41]. In another study, subjects with NAFLD were allocated to the following interventions for an 8-week period: (1) low-calorie diet (55% carbohydrates, 15% proteins, and 30% fat), (2) low-calorie/low-carb diet (45% carbohydrates, 20% protein, and 35% fat), and (3) low-calorie/low-carb and soy-rich diet (45% carbohydrates, 20% protein, and 35% fat plus 30 g of soy, in place of 30 g of red meat). The low-calorie/low-carb and soy-rich diet significantly reduced transaminases and fibrinogen compared to the other groups [42].

Malnutrition caused by protein deficiency leads to steatosis. On the other hand, excessive protein intake may cause glomerular sclerosis, hypertension, and kidney failure in persons susceptible to kidney disease [37]. In subjects with NAFLD, moderate protein intake (20–25% of total energetic value) may both be safe and have a positive impact in weight loss and improvement of IR [43]; for patients with T2DM, American Diabetes Association (ADA) suggests the prescription 15–20% of total daily energy intake from proteins [39].

### 4.4. Fats

Animal studies showed that a fat-rich diet induces steatosis and the accumulation of iron in the hepatocytes, which is favored by a high lipid intake, is a contributor for this process [44]. In obese humans, a diet with 55% fat increases the intrahepatic triglyceride levels in 35% and fasting serum insulin levels, regardless of weight [45]. Recommendations about the total amount of fat in the diet vary from 20 to 35% of total daily caloric intake [39, 40, 46].

Excessive consumption of saturated fatty acids (SFA: lard, milk, and coconut) promotes endoplasmic reticulum stress and hepatocyte injury; however, severe restriction may not be beneficial in cases of NAFLD. A study with subjects from the general subject evaluated diets with different percentages of total fat (TF) and SFA, (1) control diet (38% TF; 14% SFA), (2) National Cholesterol Education Program (NCEP) phase I diet (30% TF; 9% SFA), and (3) NCEP phase II diet (25% TF; 6% SFA), and showed that NCEP diets were related to a decrease in HDL and LDL cholesterol and an increase in triglycerides [37]. In persons with higher body fat and IR (characteristically of NAFLD), these alterations were more pronounced with NCEP phase II diet [37]. So, diets with <7% or >10% of SFA may not be adequate for NAFLD.

Monounsaturated fatty acids (MUFA), typically represented by olive oil, nuts, and avocado, may be extremely beneficial for subjects with NAFLD. Basically, they reduce LDL, oxidized LDL, and triglycerides levels; in specific populations, like diabetics, they reduce both serum glucose and VLDL and rise HDL levels. Diets in which MUFA are more than 20% of total daily caloric intake have shown benefits in NAFLD also because of the increased oxidation of other FA through activation of peroxisome proliferator-activated receptors (PPARs) alpha and gamma and reduction of lipogenesis through diminished activation of sterol regulatory element binding protein (SREBP) [37].

Persons with T2DM were randomized to four different interventions: (1) high-carb/high-fiber/low glycemic index diet, (2) high-MUFA diet, (3) high-carb/high-fiber/low glycemic index diet plus exercise, and (4) high-MUFA diet plus exercise. After eight weeks, subjects allocated to interventions 2 and 4, those with a high-MUFA diet, had significantly reduced liver fat content, independently of physical exercise levels [47].

Despite the underlying mechanism being not fully established, ingestion of large amounts of the so-called trans fatty acids (TFA, from hydrogenated oils) increases the levels of inflammatory markers and leads to endothelial dysfunction and deleterious alterations in lipid profile (increase in LDL/HDL and TC/HDL ratios). There is little evidence regarding the impact of these forms of FA in terms of hepatocyte injury and NAFLD [37]; a daily intake of <1% of calories in the form of TFA is suggested [40, 46].

For the general population and subjects with cardiovascular risk factors, recommendations regarding omega-6 polyunsaturated FA (PUFA), represented mostly by vegetable oils (soy, corn, cotton, and sunflower), ingestion are 5–10% of total daily calories and a varied ingestion of cholesterol from 200 to 300 mg/day [39, 40, 46]. Excessive ingestion of omega-6 PUFA is not encouraged because it alters the production of inflammatory markers and is more susceptible to lipid peroxidation and the consequent reduction of HDL [40]. In obese men with no liver disease who received 50 g/day of rapeseed/canola oil during four weeks, total cholesterol, LDL, and hepatic enzymes were in a healthier range than those who receive 50 g/day of olive oil [48].

Weight loss related to a hypocaloric/low-carb diet (approximately 46% of carbohydrates) seems to have a more significant impact in hepatic enzymes levels, regardless of the amount of MUFA or PUFA [49].

#### 4.4.1. Omega-3 Polyunsaturated Fatty Acids

Omega-3 PUFA supplementation has been investigated as an adjunct therapy for NAFLD with uneven results. While some authors affirm that results in humans shall be interpreted with caution [50], others say that 1 g/day of omega-3 PUFA decreases fat infiltration in the liver and that 2 g/day diminishes hepatic enzymes levels, serum fasting glucose, triglycerides, and the degree of steatosis [51]. According to AGA, supplementation with omega-3 PUFA as a specific adjunct therapy for NAFLD is not indicated because of the heterogeneity of the studies, but it may be considered to reduce high triglycerides levels in subjects with confirmed NAFLD [34]. The use of marine omega-3 (2–4 g/day) for severe hypertriglyceridemia (>500 mg/dL), which is refractory to nonpharmacological and pharmacological therapies and has increased risk of pancreatitis, is recommended [46].

### 4.5. Fibers

Fibers can be classified by their solubility. Soluble ones are represented by pectin (fruits) and gums (oat, barley, beans, peas, lentils, and chickpeas), while insoluble ones are cellulose (wheat), hemicellulose (grains), and lignin (green vegetables). They must, preferentially, come from the diet (so supplementation is not recommended if the source foods are present in the daily alimentation). Insoluble fibers increase satiety (helping in the reduction of caloric intake) and contribute to intestinal regulation; and the soluble ones slow gastric emptying and absorption of glucose and cholesterol.

Considering the high prevalence of NAFLD in patients with dyslipidemia, T2DM, and MS, the use of fibers as therapeutic strategy for glycemic, lipid, and weight controls in subjects with steatosis seems reasonable. Recommendations regarding daily fibers intake vary from 20 to 40 g/day (5–15 g/day from soluble fibers) [39, 40].

#### 4.5.1. Prebiotics and Probiotics

Prebiotics are nondigestible carbohydrates that stimulate probiotic (beneficial bacteria) growth and activity, mainly lactobacilli and bifidobacteria. Studies in animal models show promising evidence of prebiotic use in the treatment of hepatic steatosis; in humans, however, robust clinical studies are needed to determine the effects of these fibers in NASH histological markers, obesity, T2DM, and NAFLD. Furthermore, prebiotic supplementation with 30 g/day is associated with gastrointestinal side effects [52].

The definition of probiotics says it is a live microorganism that when ingested in adequate amounts promotes benefits to the host's health. They have been used to treat and prevent hepatic steatosis and these effects may be related to a variety of direct and indirect mechanisms, including modification of local microbiota, improvement of epithelial barrier, and attenuation of intestinal inflammation and oxidative stress [53].

Persons with NAFLD were randomized to consume 300 g/day of conventional yogurt or 300 g/day of probiotic enriched yogurt (*Lactobacillus acidophilus* La5 and* Bifidobacterium lactis* Bb12) for 8 weeks; the latter group had significative improvement in liver enzymes and total and LDL cholesterol levels [54]. A meta-analysis evaluating clinical trials in patients with NASH and NAFLD concluded that probiotic therapy can reduce aminotransferases and total cholesterol and attenuate IR in these populations; it is noteworthy that analyzed studies had a considerable variability regarding length of follow-up (8 weeks to 6 months), used probiotic strains, and prescribed doses [55].

A systematic review with randomized trials that evaluated the effects of probiotics prebiotics or both, in different doses, for treatment of NAFLD in adults concluded that there is no sufficient robust data to support the recommendation of their use as an alternative to NAFLD treatment [56].

### 4.6. Specific Diet Patterns: Mediterranean Diet and DASH Diet (Dietary Approaches to Stop Hypertension)

The benefits of Mediterranean diet (MeDiet) in risk factors also associated with NAFLD, as dyslipidemia, IR, T2DM, HTN, and visceral obesity, have been consolidated ever since [57] and higher Mediterranean diet scores are inversely related to ALT levels, IR, and NAFLD severity in observational studies [58]. It has been hypothesized that carotenoids, fibers, and folic acid, characteristic components of MeDiet, may have a central role in preventing or slowing oxidative stress. In addition, vegetables, important elements of the MeDiet, are important source of phytosterols, a natural cholesterol-lowering agent which reduces cardiovascular risk. MeDiet can improve adiponectin levels, a soluble matrix protein expressed by adipocytes and hepatocytes which is reduced in IR, T2DM, and obesity and linked with development of liver steatosis [59].

Clinical trials evaluating this dietary pattern specifically in patients with NAFLD are scarce [57]. One crossed randomized trial with 12 nondiabetic persons with biopsy-confirmed NAFLD evaluated the effect of Mediterranean diet in IR and hepatic steatosis. After 18 weeks of follow-up (6 for Mediterranean diet, 6 for wash-out, and 6 for control diet (low-fat)), there was no significant difference in weight loss; however, Mediterranean diet was able to reduce hepatic steatosis and improve insulin sensitivity [60]. The classic Mediterranean diet pattern is composed of fruits, vegetables, grains, nuts, moderate amounts of dairy products and fish, small amounts of poultry, red meat, and saturated fat, and emphasis on extra-virgin olive oil and red wine [37].

The DASH (Dietary Approaches to Stop Hypertension) diet is an adaptation from the USDA Dietary Guidelines and was originally tested in patients with hypertension. It is known, however, that adopting this diet pattern is highly effective in reducing the risk for fatal and nonfatal cardiovascular disease (considering coronary artery disease, stroke, and heart failure) [61]. It consists of vegetables, low-fat dairy products, fruits, integral cereals, fish, poultry, and nuts and advises against consumption of total and saturated fat, red meat, and sugars; additionally, daily sodium content is a maximum of 2.3 g/day, and the best results regarding blood pressure levels are observed with a maximum intake of 1.5 g/day [37].

It is speculated that DASH diet may be beneficial for subjects with NAFLD by directly acting on risk factors, with special emphasis for HTN, dyslipidemia, and IR. A meta-analysis evaluating randomized trials concluded that DASH diet may enhance insulin sensitivity regardless of weight loss, mainly when prescribed for more than 16 weeks [62].

### 4.7. Vitamin E Supplementation

Oxidative stress is considered one of the mechanisms of hepatocellular injury and fatty liver disease progression. For this reason, vitamin E, a known antioxidant, has been evaluated as a treatment option for NASH. Despite studies' limitations, especially regarding different doses and analyzed outcomes, it is suggested that use of vitamin E may improve steatosis, inflammation and lowers aminotransferases levels. However, no effect on liver fibrosis was observed. A randomized trial with nondiabetic 250 subjects evaluated daily supplementation with 800 IU of *α*-tocopherol for 96 weeks and demonstrated a significant reduction of the NAS score regarding lobular inflammation and liver regeneration, compared to placebo [63]. A meta-analysis of randomized trials that evaluated different doses of vitamin E in persons with liver disease concluded that supplementation may improve AST and ALT levels in cases of NASH and chronic hepatitis C, but not in NAFLD [64].

AGA suggests the use of 800 IU/day of vitamin E in nondiabetic patients with NASH to improve histological liver patterns and retard disease progression; however, its use is not recommended in diabetic subjects with NASH, in cryptogenic or NASH-related cirrhosis, or in NAFLD [34].

### 4.8. Coffee and Phenolic Compounds

Coffee is composed of more than 1,000 substances, and caffeine is the main one. Chlorogenic acids are also present (phenolic compounds from esterification of hydroxycinnamic acids (i.e., coumaric, ferulic, and quinic acid) and diterpenes, as cafestol and kahweol. Caffeine alters the transforming growth factor beta (TGF-*β*) signaling pathway, increasing SMAD proteins levels which, consequently, reduce connective tissue growth factor* (CTGF)* gene transcription and the levels of CTGF, the major stimulus for liver fibrosis [65]. Moreover, phenolic compounds present in coffee have antioxidant and hypoglycemic properties. In fact, studies have indicated an inverse relationship between coffee intake and risk of developing T2DM; a meta-analysis with observational studies and more than 1 million subjects showed that 2 cups of coffee a day decrease the risk in 12%, 2 cups of decaffeinated coffee a day reduce it in 11%, and ingestion of 200 mg/day of caffeine is related to a 14% reduction in risk for T2DM [66].

This is why coffee consumption has been evaluated in persons with NASH and NAFLD, and results indicate an inverse relationship between coffee intake and aminotransferases levels, inflammation, hepatic fibrosis, and disease progression. Nevertheless, due to heterogeneity of studies regarding daily amounts of coffee intake, method of preparation, target population, concentration of caffeine, diterpenes, and phenolic compounds of the beverages, there is no formal indication of using coffee as an adjunct therapy for NAFLD [65].

A number of beneficial effects have been attributed to other phenolic compounds present in fruits, vegetables, cocoa, teas, grapes, and wines, with special attention to resveratrol. Although studies indicate a beneficial effect of isolated polyphenols (or in extract) in the capacity of reducing liver fat accumulation, it is still necessary to establish the adequate dose and needed time for treatment of steatosis with these substances. It is noteworthy that a large number of studies showing these liver-repairing properties were in animal models [67].

Subjects with NAFLD were randomized for the intervention group (2 capsules with 150 mg of resveratrol/day) and had significant reductions of AST, ALT, and LDL cholesterol and total cholesterol levels, as well as an improvement in glucose profile compared to control group (2 capsules of placebo/day) [68]. In another study, patients with NAFLD were allocated to control (placebo) or intervention (500 mg of resveratrol/day); both had recommendation of balanced diet and increase of exercise levels. Individuals in the intervention group (lifestyle modification and resveratrol supplementation) had significant improvement in steatosis, liver enzymes, and markers of apoptosis and inflammation [69].

#### 4.8.1. Alcohol Consumption

Epidemiologic studies have shown a protective effect of light to moderate daily alcohol consumption on the development and progression of NAFLD, primarily through the improvement in peripheral IR [70]. Light (40–140 g of alcohol/week) and moderate alcohol intake (140–280 g/week) have been associated with lower prevalence of hepatic steatosis [71] and lower levels of serum transaminases activity [72]. Meta-analysis involving 43,175 adults (modest drinkers defined as <40 g alcohol/day) showed that moderate consumption of alcohol was associated with 31% lower risk of having NAFLD and 50% lower risk of developing an advanced disease stage [73].

In patients previously diagnosed with NAFLD, modest alcohol ingestion was associated with a lesser degree of severity of NASH as well as of fibrosis when nondrinkers were compared to modest drinkers with a normal BMI; after adjustment for other variables, individuals who had moderate alcohol intake showed 44% reduced odds of having a diagnosis of NASH, lower odds for fibrosis (OR 0.56, 95% CI 0.41–0.77), and ballooning hepatocellular injury (OR 0.66, 95% CI 0.48–0.92) [74]. However, those individuals who have underlying hepatic steatosis or NASH should not use ethanol chronically since the data available do not support a beneficial effect of alcohol in this situation [70].

#### 4.8.2. Summary of Nutritional Recommendations

A summary of the main diet recommendations for NAFLD management can be obtained as follows:

 Total caloric intake (TCI): adjusted for weight loss, if necessary,
   ↓ 3 to 5% of the body weight in steatosis (HS),
   ↓ 10% of the body weight in steatohepatitis (NASH),
   ↓ 500–1,000 kcal/day,
   Mediterranean or DASH dietary patterns.
 Carbohydrates: 45 to 60% of TCI, preferably not refined,
   ↓ 45% to accelerate weight loss (6 months).
 Fibers: 20 to 40 g/day (5 to 15 g of soluble fiber).
 Total fat: 20 to 35% of TCI.
 Saturated fats: 7 to 10% of TCI.
 Polyunsaturated fats: 5 to 10% of TCI.
 Monounsaturated fats: 15 to 20% of TCI.
 Omega-3: preferably through the weekly intake of fish,
   2 to 4 g/day (supplement of oil fish) if serum triglycerides ≥ 500 mg/dL.
 Dietary cholesterol: 200 to 300 mg/day.
 Trans fatty acids: <1% of TCI.
 Protein: 20% of TCI.
 Vitamin E: 800 IU/day (supplement) in NASH.
 Daily and moderate coffee consumption.

DASH stands for Dietary Approaches to Stop Hypertension.

## 5. Physical Exercise and NAFLD

The use of exercise in the prevention and treatment of NAFLD has already been established, as it acts in different pathways involved in the pathophysiology of such condition [75].

The first pathway is through the effects of exercise on classic risk factors, mainly obesity and alterations in lipid and glucose metabolism. Exercise also acts in conjunction with diet modifications, leading to attenuation of NAFLD through weight loss. Previous studies demonstrated that patients with steatosis treated with a combination of diet and exercise, and loss of 5% of body weight, had a reduction in aminotransferases levels and sustained these reduction for at least 15 months after treatment [76, 77].

On the other hand, some studies were focused on the direct effects of exercise over NAFLD, independently of weight loss [78–81]. A study showed that both low- and moderate-intensity aerobic exercise reduced hepatic enzymes levels and improved insulin sensitivity in patients with NAFLD, after three months, regardless of weight changes [80].

Comparing the effect of three different exercise protocols (aerobics, resistance, and aerobics + resistance) visceral and hepatic fat, liver enzymes, and IR (according to the HOMA-IR index) in overweight adults, Slentz et al. also demonstrated that aerobic exercise was more effective, for it leads to significant reductions in abdominal (total and subcutaneous), visceral, and hepatic fat, ALT and HOMA-IR [82]. The first evidence that regular practice of aerobic exercise could,* per se*, reduce the amount of hepatic lipids in obese subjects was showed in a study using protons spectroscopy with magnetic resonance to verify the hepatic amount of triglyceride. After four weeks of aerobic exercise, with no significant weight change, this group of sedentary and obese volunteers had significant reductions of visceral adipose tissue (VAT) volume, hepatic triglycerides levels, and serum free fatty acids, showing that exercise may have beneficial effects on the liver independently of weight loss [79].

The same was demonstrated for resistance exercise. Hallsworth et al. demonstrated that regular practice of resistance exercise (3 times a week, for 8 weeks) was effective in reducing intrahepatic lipids in subjects with NAFLD [81]. Besides, this kind of physical activity helped to improve muscle mass and strength and insulin sensitivity in that group.

The main physiological mechanisms involved in the action of aerobic exercise in NAFLD encompass activation of adenosine monophosphate kinase (AMPK) protein, with the subsequent reduction of malonyl coenzyme A (malonyl CoA), which allows an enhanced action of carnitine acyl transferase 1 (CAT1), improving mitochondrial oxidation and transport of fatty acids [82, 83].

The mechanisms behind the reduction of intrahepatic lipids that accompanies physical exercise also seem to reflect changes in insulin sensitivity. Elevated circulating insulin levels lead to SREBP transcription factors “overexpression,” mainly hepatic SREBP-1c, stimulating* de novo* lipogenesis and increasing intrahepatic lipids [83, 84].

In subjects with NAFLD,* de novo* lipogenesis is continuously high, which contributes to intrahepatic lipid accumulation and elevated circulating triglycerides levels. This, in turn, leads to disease exacerbation for creating a vicious cycle in which the high intrahepatic lipid concentration inhibits insulin action in the liver, leading to high portal levels of insulin and increase in intrahepatic lipids [84]. There is data suggesting that physical exercise (both aerobics and resistance) may break this cycle by improving glucose control and lipid oxidation through augmentation of GLUT-4 glucose transporter in striate muscle, expression and activity of glycogen synthase enzyme, insulin receptors, and glycogen storage in muscle and liver [81, 85].

Therefore, resistance exercise seems to have a beneficial effect on NAFLD by enhancing circulating FA and glucose uptake, thus reducing the impact of hepatic insulin-stimulated* de novo* lipogenesis. On the other hand, aerobic exercise seems to increase muscular intracellular synthesis of triglycerides, decrease FA metabolites accumulation, and suppress the inflammatory state associated with IR [86, 87].

Another way by which exercise may be beneficial for patients with NAFLD is attenuation of the inflammatory state. This is, in part, due to the action of myokines (cytokines and other peptides produced and secreted by muscle fibers) which have paracrine and endocrine effects [88]. These substances are released with muscle contraction and may exert both direct and indirect (acting in fat metabolism) anti-inflammatory effects [88, 89].

The first myokine to be studied was interleukin-6 (IL-6), produced in response to contraction of oxidative and glycolytic muscle fibers [89]. In striate muscle, IL-6 acts through activation of AMPK and/or phosphatidylinositol-3-kinase (PI3K) increasing lipid oxidation and muscular glucose consumption; in fat tissue, it plays an important role in fat metabolism, increasing lipolysis; and in the liver, it increases gluconeogenesis [88, 89]. Therefore, differently from the proinflammatory action of IL-6 in obese subjects, when produced by muscle fiber in response to exercise it can have anti-inflammatory properties. In persons with abnormal liver function, exercise helps reducing high serum levels of ferritin and thiobarbituric acid-reactive substances (TBARS) and increasing adiponectin concentrations [90]. Considering that high levels of ferritin and TBARS are markers of hepatic inflammation and exacerbated lipid peroxidation, these findings show that exercise attenuates the inflammatory process and helps in the improvement of innate immunity and in balancing the proinflammatory signaling pathways in obese persons with NAFLD [90, 91]. Reduction of proinflammatory cytokines concentrations, as tumor necrosis factor alpha (TNF-*α*) and ROS, also attenuates oxidative stress and hepatic inflammation [90].

Although it is clear that each session of physical exercise induces an anti-inflammatory environment, additional research is needed to understand which type of exercise is more effective in controlling NAFLD-associated inflammation. To date, the adequate dose (intensity and load) of exercise is not determined once available studies used a large number of different and short-term protocols, which prevent the establishment of a consensual recommendation. Thereby, recent guidelines about NAFLD treatment indicate the following regarding exercise prescription [75, 78, 92]:

(i) 20 to 60 minutes (or more) of moderate intensity (45%–70% VO_2_ max) aerobic exercise, using large muscle groups, at least 5 days a week.
(ii) Moderate-high intensity resistance exercise 3 times a week.
(iii) For additional indirect benefits related to weight loss, physical exercise more than 250 minutes a week.

These aims can be achieved through different types of activities, but all subjects should have an objective evaluation so possible contraindications may be identified and the appropriate exercise intensity may be prescribed. In general, available data support physical exercise, mainly aerobics, as beneficial for NAFLD. However, broader clinical trials are necessary to better define the role of resistance exercise and combination of different exercise modalities so an ideal exercise protocol can be established.

## 6. Final Considerations

Treatment of NAFLD implies multidisciplinary care. Combined therapies using exercise, medications, and dietetic interventions can be more effective than each prescription alone. Individualized approaches are important to increase adhesion to treatment.

## Figures and Tables

**Table 1:** Clinical *signs and symptoms* and laboratory picture of NAFLD.

Frequently asymptomatic	High ALT (2–4x)
Vague and unspecific symptoms	High GGT (2–6x)
Abdominal right upper quadrant discomfort	Mild elevation of AST (can be elevated in cirrhosis)
Fatigue	
Dyspepsia	
Overweight (BMI ≥ 25 kg/m^2^)	Blood glucose > 100 mg/dL
High blood pressure	Triglycerides > 150 mg/dL
Central adiposity	Total cholesterol > 200 mg/dL
Hepatomegaly (in 50%)	LDL > 130 mg/dL
Splenomegaly (in 25%)	HDL < 45 mg/dL

NAFLD: nonalcoholic fatty liver disease; BMI: body mass index; ALT: alanine aminotransferase; GGT: gamma-glutamyl transferase; AST: aspartate aminotransferase; LDL: low density lipoproteins; HDL: high density lipoprotein.
